# Methylthioadenosine Suppresses Salmonella Virulence

**DOI:** 10.1128/IAI.00429-18

**Published:** 2018-08-22

**Authors:** Jeffrey S. Bourgeois, Daoguo Zhou, Teresa L. M. Thurston, James J. Gilchrist, Dennis C. Ko

**Affiliations:** a Department of Molecular Genetics and Microbiology, School of Medicine, Duke University, Durham, North Carolina, USA; b University Program in Genetics and Genomics, Duke University, Durham, North Carolina, USA; c Department of Biological Sciences, Purdue University, West Lafayette, Indiana, USA; d Section of Microbiology, Centre for Molecular Microbiology and Infection, Imperial College London, London, United Kingdom; e Wellcome Trust Centre for Human Genetics, University of Oxford, Oxford, United Kingdom; f Department of Paediatrics, University of Oxford, Oxford, United Kingdom; g Division of Infectious Diseases, Department of Medicine, School of Medicine, Duke University, Durham, North Carolina, USA; University of California San Diego School of Medicine

**Keywords:** SPI-1, Salmonella, flagellar motility, inflammation, metJ, metabolism, methionine salvage, methylthioadenosine, virulence regulation

## Abstract

In order to deploy virulence factors at appropriate times and locations, microbes must rapidly sense and respond to various metabolite signals. Previously, we showed a transient elevation of the methionine-derived metabolite methylthioadenosine (MTA) concentration in serum during systemic Salmonella enterica serovar Typhimurium infection. Here we explored the functional consequences of increased MTA concentrations on *S*. Typhimurium virulence. We found that MTA, but not other related metabolites involved in polyamine synthesis and methionine salvage, reduced motility, host cell pyroptosis, and cellular invasion. Further, we developed a genetic model of increased bacterial endogenous MTA production by knocking out the master repressor of the methionine regulon, *metJ*. Like MTA-treated *S*. Typhimurium, the Δ*metJ* mutant displayed reduced motility, host cell pyroptosis, and invasion. These phenotypic effects of MTA correlated with suppression of flagellar and Salmonella pathogenicity island 1 (SPI-1) networks. *S*. Typhimurium Δ*metJ* had reduced virulence in oral and intraperitoneal infection of C57BL/6J mice independently of the effects of MTA on SPI-1. Finally, Δ*metJ* bacteria induced a less severe inflammatory cytokine response in a mouse sepsis model. Together, these data indicate that exposure of *S*. Typhimurium to MTA or disruption of the bacterial methionine metabolism pathway suppresses *S*. Typhimurium virulence.

## Abstract (precis)

In order to deploy virulence factors at appropriate times and locations, microbes must rapidly sense and respond to various metabolite signals. Previously, we showed a transient elevation of the methionine-derived metabolite methylthioadenosine (MTA) concentration in serum during systemic Salmonella enterica serovar Typhimurium infection.

## INTRODUCTION

Microbial communities within a mammalian host are bombarded by an array of intercellular, interspecies, and cross-kingdom metabolites and proteins. Cross-kingdom signaling plays important roles in Salmonella pathogenesis. For instance, in order to invade nonphagocytic host cells, Salmonella must deploy a secretion system encoded by Salmonella pathogenicity island 1 (SPI-1) (1, 2), which is regulated by many signals, such as pH, bile, and short-chain fatty acids (3–7). Together, these factors spatially limit the bacteria so that most invasion occurs in the ileum (8). Furthermore, recent work demonstrates that a host mimic of the bacterial AI-2 quorum molecule can directly impact Salmonella enterica serovar Typhimurium gene expression *in vitro* by activating the *lsr* operon (9). Understanding how the bacterium's environment influences Salmonella pathogenesis is important, as it could help to inform future therapeutic interventions to suppress virulence.

One signal that may facilitate cross talk between host and pathogen during infection is methylthioadenosine (MTA), a key metabolite in methionine metabolism. In addition to its role in protein synthesis, methionine is used in both eukaryotic and prokaryotic systems to generate *S*-adenosylmethionine (SAM), which is a critical methyl donor for a number of reactions (10, 11). SAM catabolism results in a number of metabolic by-products, including MTA and *S*-adenosylhomocysteine (SAH). In many eukaryotic and prokaryotic systems, MTA is recycled back into methionine; however, Escherichia coli and *S*. Typhimurium cannot salvage methionine from MTA (12). Instead, E. coli and Salmonella spp. regulate intracellular MTA concentrations by using an MTA/SAH nucleosidase (*pfs*) to cleave MTA into 5′-methylthioribose and excreting it (13, 14). MTA regulation is considered critical for the bacterial cell, as deletion of *pfs* impairs growth (15), but the effects of MTA on Salmonella virulence remain unknown.

Previously, our labs determined that MTA plays a multifaceted role in Salmonella infection. We originally identified MTA to be a positive regulator of host cell pyroptosis, a rapid, proinflammatory form of cell death, during Salmonella infection (16). More recently, we showed that host MTA is released into plasma during *S*. Typhimurium infection and that high plasma MTA levels are associated with poor sepsis outcomes in humans (17). Paradoxically, we showed that treatment of mice with exogenous MTA suppresses sepsis-associated cytokines and extends the life span of mice infected with a lethal dose of *S*. Typhimurium (17). While this finding was consistent with previous reports that MTA acts as an anti-inflammatory molecule (18–20), it was in contrast to our findings that MTA primes cells to undergo pyroptosis. Together, these data led us to hypothesize that increased extracellular concentrations of MTA could potentially have independent effects on both the host and the pathogen during infection.

Here we show that fluctuations in MTA levels regulate *S*. Typhimurium virulence *in vitro* and *in vivo*. Treatment of *S*. Typhimurium with exogenous MTA prior to infection or increasing endogenous bacterial production of MTA through genetic deletion of the methionine regulon suppressor, *metJ*, reduced the induction of pyroptosis and invasion *in vitro*. Furthermore, we report that both Δ*metJ* mutants and MTA-treated bacteria demonstrate transcriptional, translational, and functional reductions in motility and SPI-1 activity. Finally, we found that Δ*metJ* mutants have reduced virulence *in vivo* and that disrupting the methionine metabolism pathway in the bacteria can influence the inflammatory state of the host. Together, these data reveal the importance of MTA and bacterial methionine metabolism in regulating *S*. Typhimurium virulence and host inflammation and provide a possible example of host-pathogen metabolite cross talk during infection.

## RESULTS

### Exogenous MTA reduces the ability of *S*. Typhimurium to induce pyroptosis and invade host cells *in vitro*.

**FIG 1:**
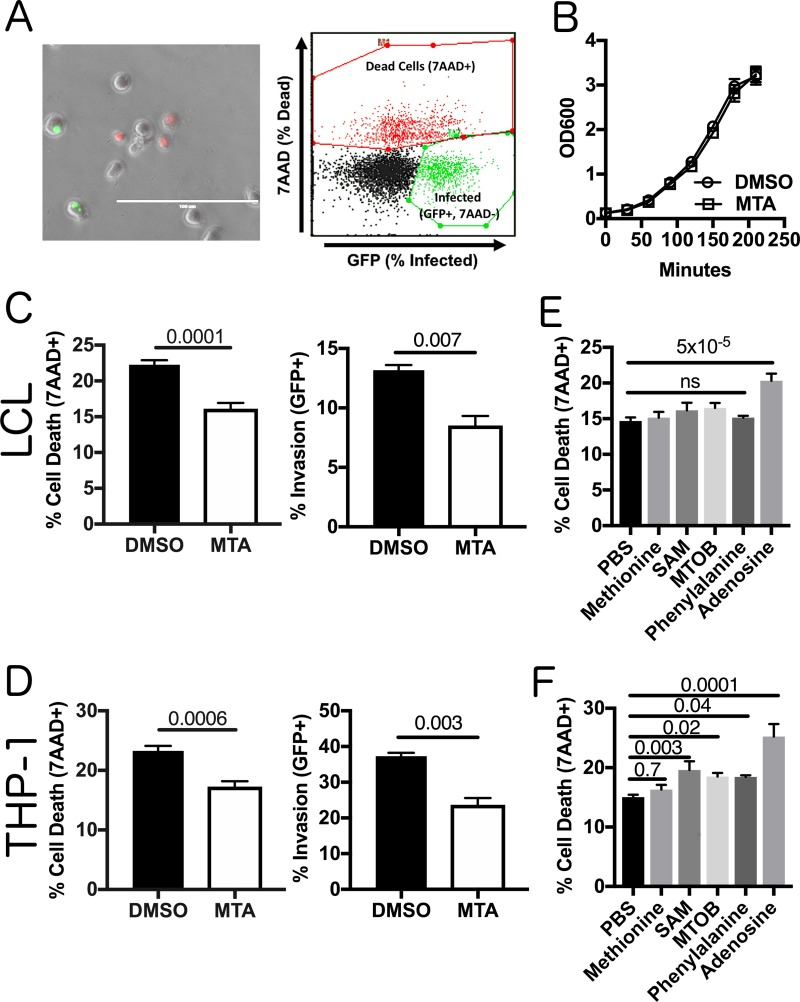
MTA treatment of *S*. Typhimurium reduces pyroptosis and invasion *in vitro*. (A) A modified gentamicin protection assay using an inducible GFP plasmid was used to detect pyroptosis (7-AAD^+^ red nuclear staining) and host cell invasion (intracellular GFP^+^ bacteria). This is observable by fluorescence microscopy and quantifiable by flow cytometry. Bar, 100 μm. (B) Exogenous MTA has no effect on the growth of *S*. Typhimurium in rich medium. The optical density at 600 nm (OD_600_) for *S*. Typhimurium treated with 300 μM MTA or 0.5% DMSO was measured every 30 min and showed that it exhibited equivalent growth with both treatments (*n* = 3). (C, D) Treatment of bacteria with 300 μM MTA during growth to late log phase (2 h 40 min) reduced pyroptosis (MOI, 30) and host cell invasion (MOI, 10) in LCLs measured at 3 h postinfection in 18592 LCLs (C) and THP-1 cells (D). Percent cell death represents all 7-AAD-positive cells under each infected condition, with the baseline uninfected cell death being subtracted. See the gating in panel A. Data were normalized to the global mean across five experiments, and *P* values, indicated at the top, were generated by a Student's *t* test. (E, F) Treating bacteria with other methionine-related metabolites (methionine, SAM, MTOB, and phenylalanine) or adenosine does not suppress host cell pyroptosis (MOI, 30), based on 3 to 5 independent experiments in 18592 LCLs (E) or THP-1 cells (F). Data were normalized to the global mean, and *P* values were generated by a one-way analysis of variance with Dunnett's multiple-comparison test. All error bars represent the standard error of the mean. ns, not significant.

Previously, we demonstrated elevated concentrations of MTA in plasma during systemic infection of mice with *S*. Typhimurium (17). While the effects of MTA on the host inflammatory response have been documented (16–20), we asked whether elevated extracellular MTA levels could directly impact bacterial virulence. We examined the ability of *S*. Typhimurium pretreated with MTA (300 μM) to induce pyroptosis and invade human cells. As our original studies that identified MTA to be a modulator of pyroptosis were performed in lymphoblastoid cells (LCLs) and because B cells are a natural target of *S*. Typhimurium invasion *in vivo* (21–23), we first looked for an effect of exogenous MTA in 18592 LCLs. We assessed both pyroptosis and invasion by pairing a modified gentamicin protection assay with flow cytometry, as previously described (24) (Fig. 1A). Briefly, pyroptosis was measured by quantifying the number of cells that stained positive for 7-aminoactinomycin D (7-AAD) at 3 h postinfection with *S*. Typhimurium. Independently, cellular invasion was quantified by using bacteria with a green fluorescent protein (GFP)-harboring plasmid, inducing GFP production after gentamicin treatment, and quantifying the number of GFP-positive (GFP^+^), 7-AAD-negative (7-AAD^−^) host cells at 3 h postinfection. MTA pretreatment had no effect on bacterial growth from the initial overnight culture dilution through late log phase to induce SPI-1 gene expression (Fig. 1B). MTA-pretreated bacteria displayed a 30% decrease (*P* = 0.0001) in their ability to induce pyroptosis (Fig. 1C). Furthermore, we observed a 35% decrease (*P* = 0.007) in their ability to invade LCLs (Fig. 1C). We observed similar effects of MTA on pyroptosis and invasion in THP-1 monocytes (Fig. 1D). This was in contrast to our previous finding that MTA treatment of host cells primes them to undergo higher levels of pyroptosis upon *S*. Typhimurium infection (16) and demonstrates that the molecule has separate effects on both the host and the pathogen.

This reduction in pyroptosis could not be reproduced by treating the bacteria with other metabolites related to the mammalian methionine salvage pathway, including methionine, SAM (also known as AdoMet), α-keto-γ-(methylthio)butyric acid (MTOB), and phenylalanine (Fig. 1E and F). Similarly, adenosine (which lacks only the methylthio group of MTA) was not sufficient to suppress pyroptosis induction. In fact, adenosine increased pyroptosis in both LCLs and THP-1 monocytes. Of note, the bacterial cell is reportedly impervious to SAM (25, 26), so we cannot rule out the possibility that high intracellular concentrations of the molecule could suppress pyroptosis; however, our results rule out the possibility that the molecule is an external signal regulating pyroptosis. Thus, these data demonstrate that MTA exposure uniquely suppresses the ability of *S*. Typhimurium to induce pyroptosis and invade host cells.

### *metJ* deletion in *S*. Typhimurium elevates the levels of MTA.

**FIG 2:**
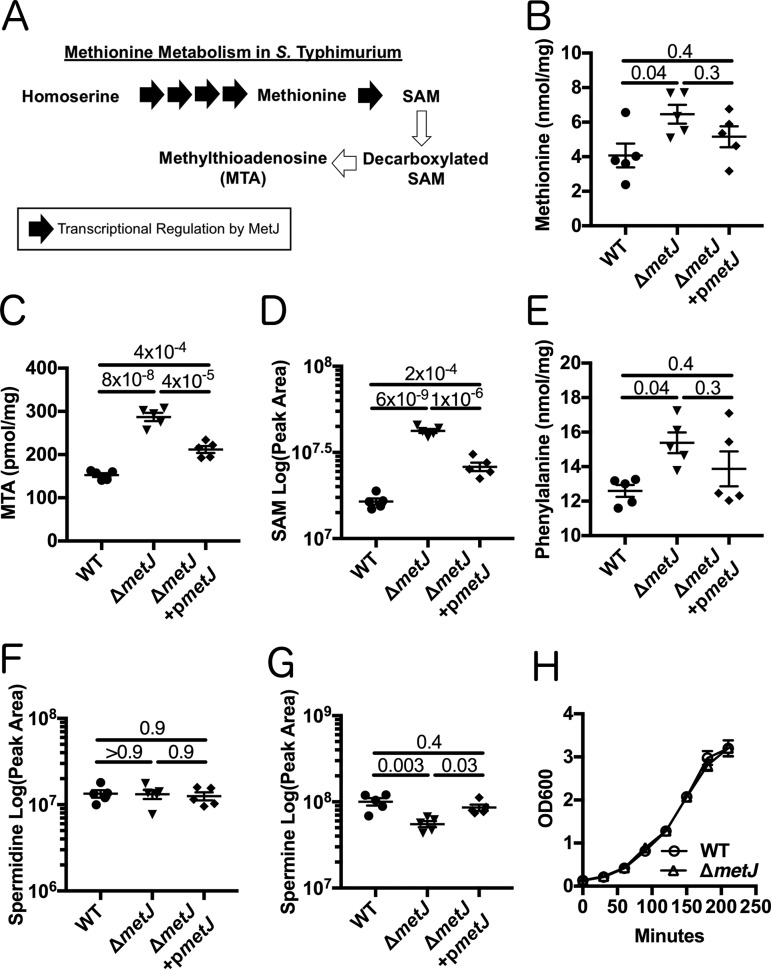
*metJ* deletion results in disruption of methionine metabolism. (A) MetJ regulates the generation of methionine and SAM in *S*. Typhimurium by transcriptionally repressing the methionine regulon. Black arrows represent enzymes transcriptionally repressed by MetJ. (B to E) Deletion of *metJ* leads to increased methionine (B), MTA (C), SAM (D), and phenylalanine (E) levels, as measured by mass spectrometry (*n* = 5 biological replicates). (F, G) *metJ* deletion did not affect spermidine concentrations (F) but did result in decreased amounts of spermine (G). *P* values, indicated at the top, were generated through a one-way analysis of variance with Tukey's multiple-comparison test. (H) *metJ* deletion did not affect bacterial growth in LB (*n* = 3 biological replicates grown in LB plus 0.5% DMSO). All error bars represent the standard error of the mean.

In order to provide independent evidence for MTA-mediated regulation of Salmonella virulence, we genetically disrupted the *S*. Typhimurium methionine metabolism pathway. The protein MetJ is the master repressor of the methionine regulon and transcriptionally blocks multiple enzymatic steps that enable the generation of methionine and SAM (Fig. 2A) (10). We hypothesized that deletion of *metJ* would relieve this transcriptional suppression and result in elevated intracellular MTA levels. Therefore, we generated a Δ*metJ* mutant and performed mass spectrometry to examine how metabolites in the methionine metabolism pathway were impacted by the mutation. In line with previous reports, we observed an increase in methionine levels in the Δ*metJ* mutant (27) (Fig. 2B). MTA, SAM, and phenylalanine levels were also increased (Fig. 2C to E). Expression of *metJ* from a plasmid reversed these increases (Fig. 2B to E). Consistent with MTA being an inhibitor of polyamine synthesis, the polyamine spermine level was decreased in the Δ*metJ* mutant, while no change was observed for another polyamine, spermidine (Fig. 2F and G). Disrupting the methionine metabolism pathway by deleting *metJ* did not affect bacterial growth (Fig. 2H).

### Elevated endogenous MTA suppresses pyroptosis and invasion *in vitro*.

**FIG 3:**
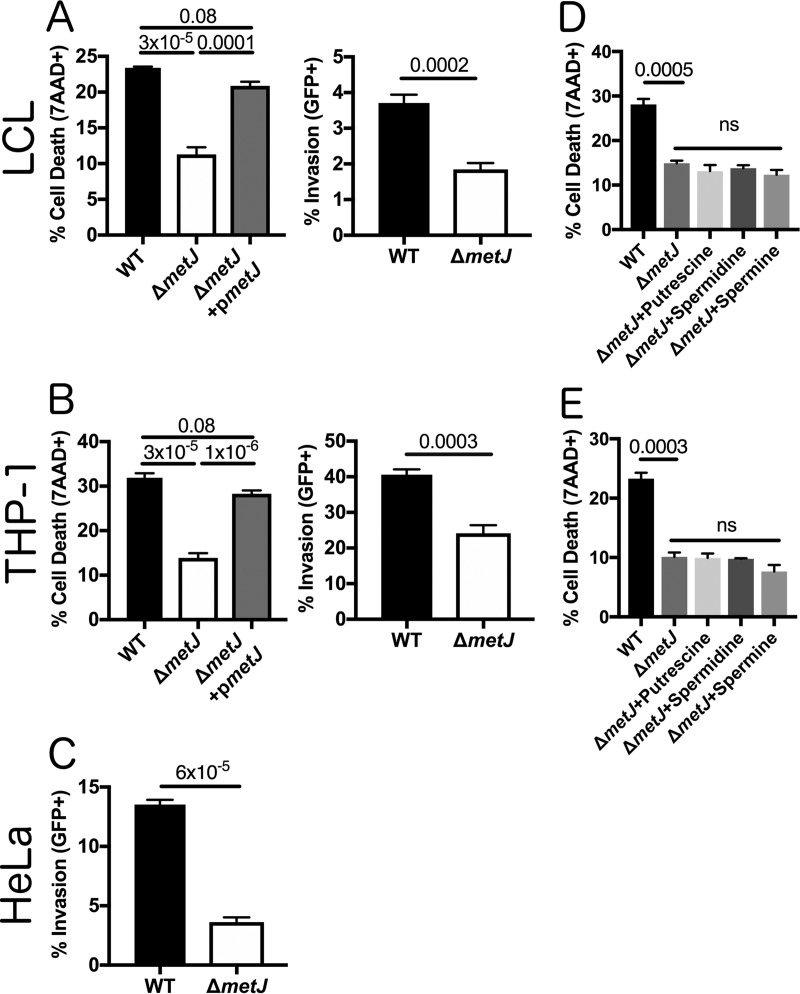
*metJ* deletion reduces pyroptosis and invasion *in vitro*. (A) Deletion of *metJ* reduces pyroptosis and invasion of 18592 LCLs. (B) Deletion of *metJ* reduces pyroptosis and invasion of THP-1 monocytes. (C) Deletion of *metJ* reduces invasion of HeLa cells. For the assays whose results are presented in panels A, B, and C, pyroptosis and invasion were measured at 3 h postinfection using a modified gentamicin protection assay across at least three independent experiments. Percent cell death represents all 7-AAD^+^ cells under each infected condition, with the baseline uninfected cell death being subtracted. See the gating in Fig. 1A. Data were normalized to the global mean, and *P* values, indicated at the top, were calculated either through a one-way analysis of variance with Tukey's multiple-comparison test or by a Student's *t* test. (D, E) Suppression of pyroptosis could not be rescued by treating bacteria with polyamines. Bacteria were treated with 300 μM putrescine, spermidine, or spermine for 2 h 40 min prior to infection to determine whether the effects on pyroptosis were due to effects on polyamine synthesis. For all experiments with LCLs or THP-1 monocytes, cells were infected at an MOI of 30. HeLa cells were infected at an MOI of 5. For the assays whose results are presented in panels D and E, data were generated from two independent experiments and normalized to the global mean, and *P* values were generated by a one-way analysis of variance with Dunnett's multiple-comparison test. Error bars represent the standard error of the mean.

After demonstrating that *metJ* deletion leads to the accumulation of MTA in the bacterial cell, we examined whether critical Salmonella virulence processes are repressed in the Δ*metJ* mutant. Similar to *S*. Typhimurium treated with MTA, the Δ*metJ* mutant had a reduced ability to induce pyroptosis in LCLs and THP-1 monocytes (Fig. 3A and B). Importantly, MTA was the only metabolite whose levels were measured to be elevated in the Δ*metJ* mutant that inhibited the levels of pyroptosis when added exogenously to wild-type (WT) bacteria (Fig. 1D). This reduction in pyroptosis was rescued by expressing *metJ* from a plasmid. *S*. Typhimurium Δ*metJ* had reduced invasion of LCLs, THP-1 monocytes, and HeLa cells (Fig. 3A to C). Spermine and other polyamines could not rescue the pyroptosis induced by *S*. Typhimurium Δ*metJ*, suggesting that these findings are not mediated by the reduction in spermine that we observed in the Δ*metJ* mutant (Fig. 3D and E).

### Disruption of methionine metabolism and treatment with exogenous MTA impairs *S*. Typhimurium motility.

Motility and the SPI-1 type III secretion system (T3SS) are critical processes for the induction of pyroptosis and *S*. Typhimurium invasion *in vitro*. Motility increases the frequency by which interactions between host and *S*. Typhimurium cells occur and enables bacterial scanning of the host cell surface to optimize invasion (28, 29). The SPI-1 secretion system not only enables the transport into the host cell of effector proteins that enable invasion (1, 2) but also acts as a trigger for pyroptosis in human cells (30–34). Therefore, our observation that both pyroptosis and invasion are suppressed in MTA-treated *S*. Typhimurium and *S*. Typhimurium Δ*metJ* led us to hypothesize that motility and/or SPI-1 are suppressed in response to increased concentrations of MTA.

**FIG 4:**
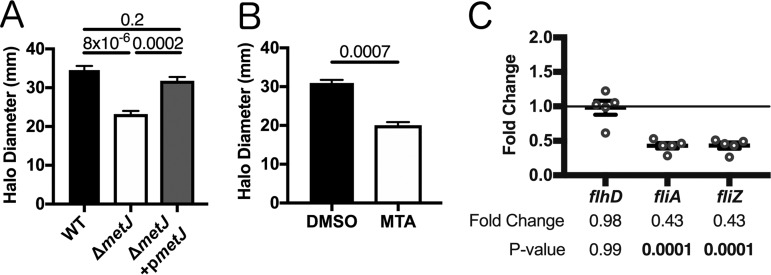
*metJ* deletion and MTA treatment of *S*. Typhimurium reduces motility. (A) *S*. Typhimurium motility is suppressed in Δ*metJ* mutants. Motility was measured after 6 h at 37°C on 0.3% LB agar. (B) Exogenous MTA suppresses *S*. Typhimurium motility. Motility was measured after 6 h on 0.3% LB agar with 0.5% DMSO or 300 μM MTA. Data from the assays whose results are presented in panels A and B represent those from at least three independent experiments normalized to the global mean, and *P* values, indicated at the top, were calculated by a one-way analysis of variance with Tukey's multiple-comparison test or a Student's *t* test. (C) Flagellar genes are suppressed in the Δ*metJ* mutant. RNA was extracted from wild-type *S*. Typhimurium or Δ*metJ* mutant bacterial cultures grown to late log phase in LB broth (*n* = 5) and analyzed by qPCR, with the ribosomal *rrs* gene serving as the endogenous control. Each dot represents an independent biological replicate. *P* values were calculated by a one-way analysis of variance with Dunnett's multiple-comparison test.

In order to determine whether motility was impaired in the Δ*metJ* mutant, we performed a standard bacterial soft agar motility assay (35) and found that the Δ*metJ* mutant was able to traverse only two-thirds of the distance of the wild-type bacteria over 6 h (Fig. 4A). This motility defect was restored by expression of *metJ* from a plasmid. In order to confirm that increased MTA levels were sufficient to drive this phenotype, we also examined *S*. Typhimurium motility on soft agar containing 300 μM MTA. Like the Δ*metJ* mutant, wild-type bacteria swimming on MTA-containing agar demonstrated approximately two-thirds the motility of those on dimethyl sulfoxide (DMSO)-containing agar (Fig. 4B). To further characterize this motility defect, we examined the expression of key motility regulators in the Δ*metJ* mutant by quantitative PCR (qPCR). While the master regulator of the flagellar regulon, *flhD*, did not show reduced expression, two class 2 flagellar genes, *fliA* and *fliZ*, showed significant downregulation, which correlates with the decrease in motility (Fig. 4C). Together, these data reveal that MTA suppresses the *S*. Typhimurium flagellar regulon downstream of *flhD* transcription, resulting in impaired motility *in vitro*.

### Elevated endogenous MTA suppresses expression of genes carried on SPI-1.

Deploying the SPI-1-encoded T3SS depends on a complex regulatory network in which HilD, HilC, and RtsA drive expression of *hilA* (36, 37). HilA then enables the expression of the *inv-spa* and *prg-org* operons (38–40). Both HilD and HilA directly promote the expression of the first gene in the *inv-spa* operon, *invF* (41), which is then able to drive the expression of the *sic-sip* operon and a number of other critical effectors (39, 42, 43).

**FIG 5:**
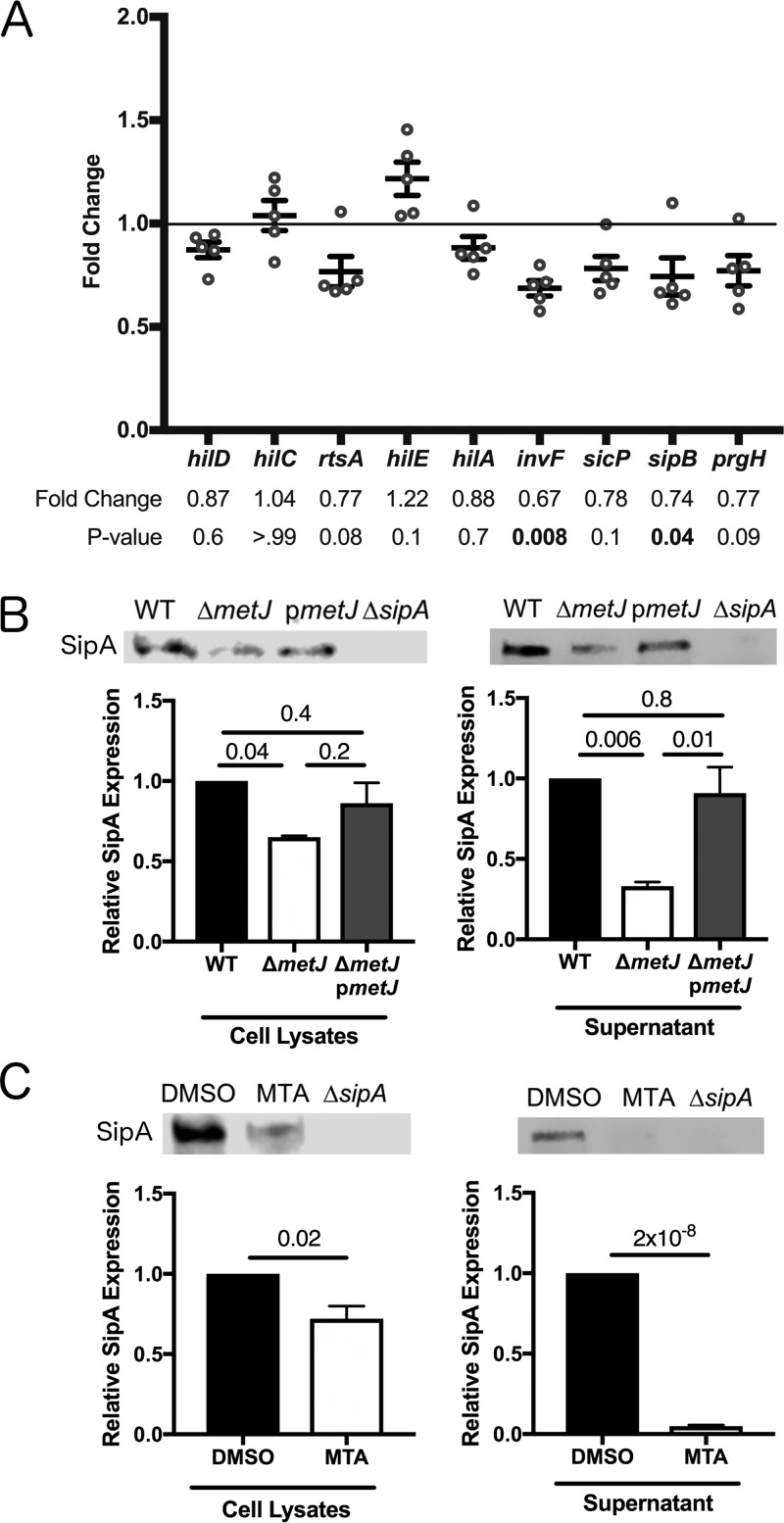
*metJ* deletion and MTA treatment of *S*. Typhimurium reduce SPI-1 secretion. (A) SPI-1 genes are suppressed in Δ*metJ* mutants. RNA was extracted from wild-type *S*. Typhimurium or Δ*metJ* mutant bacterial cultures grown to late log phase in LB broth (*n* = 5) and analyzed by qPCR, with the ribosomal *rrs* gene serving as the endogenous control. Each dot represents an independent biological replicate. *P* values, indicated at the top, were calculated by a one-way analysis of variance with Dunnett's multiple-comparison test. (B) SipA secretion is suppressed in Δ*metJ* mutants. The SipA protein was measured by Western blotting of cell lysates or cell-free supernatants collected at late log phase of growth. (C) SipA secretion is suppressed in *S*. Typhimurium treated with exogenous MTA. SipA protein was measured by Western blotting of cell lysates or cell-free supernatants collected at late log phase of growth from bacteria grown in either 0.5% DMSO or 300 μM MTA. For panels E and F, cell lysates were normalized by the total protein content. Cell-free supernatants were spiked with 100 ng/μl of BSA as a loading control, concentrated by TCA precipitation, and normalized by volume and total protein. Data represent those from three independent experiments and are normalized to wild-type expression. *P* values were calculated by a one-way analysis of variance with Tukey's multiple-comparison test or a Student's *t* test. All error bars represent the standard error of the mean.

Three lines of evidence demonstrated suppression of SPI-1 by MTA. First, we observed suppression of genes carried by SPI-1 in *S*. Typhimurium Δ*metJ* by qPCR. In particular, we saw a decrease in *invF* expression, as well as a decrease in expression of the translocon component *sipB*. The finding that *sipB* is significantly suppressed is of note, as SipB is involved in induction of host cell death by *S*. Typhimurium (33). Further, while not statistically significant when taking into account multiple-test correction, we saw comparable changes trending toward significance in the expression of the regulatory factor *rtsA*, the chaperone protein *sicP*, and the needle complex component *prgH* (Fig. 5A). This shows that there is at least modest suppression of SPI-1-regulated genes by MTA on the transcriptional level under standard culture conditions. Second, reduced expression of SipA, a SPI-1-secreted effector, was detected in Δ*metJ* mutant cell lysates by Western blot staining (Fig. 5B). Third, this reduction in SipA was greater in Δ*metJ* mutant cell-free supernatants than cell lysates, suggesting that both expression of SipA and its secretion by the T3SS apparatus are suppressed in the mutant (Fig. 5B). This is consistent with the suppression *of invF* and *sipB* observed by qPCR. These reductions in SipA expression were also detected in MTA-treated *S*. Typhimurium (Fig. 5C). Together, these data demonstrate suppression of the SPI-1 network by MTA.

### Pyroptosis, invasion, and motility are independently disrupted in the Δ*metJ* mutant.

Our results indicated that both the flagellar regulon and SPI-1 secretion are disrupted in the Δ*metJ* mutant. The flagellar regulon helps control SPI-1 gene expression through FliZ-mediated posttranslational HilD regulation (44–46). Likewise, HilD binds the *flhDC* promoter to activate the flagellar regulon (47, 48). Therefore, our results were compatible with either MTA suppressing the flagellar regulon to suppress the SPI-1 pathway, MTA suppressing the SPI-1 pathway to suppress the flagellar regulon, or MTA suppressing both pathways through independent mechanisms.

**FIG 6:**
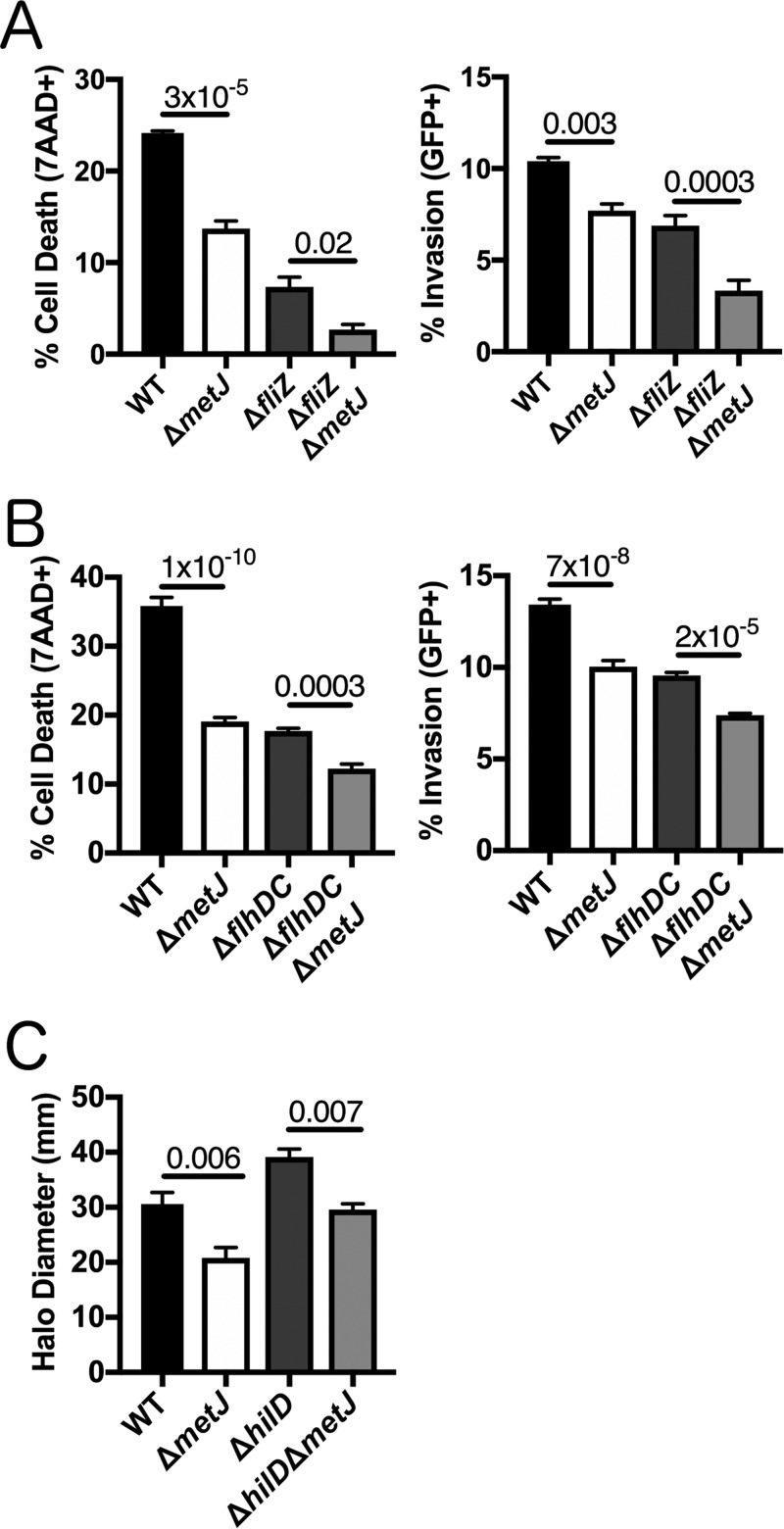
*metJ* deletion suppresses pyroptosis and invasion independently of the flagellar regulon. (A) Reductions in pyroptosis and invasion in the Δ*metJ* mutant do not depend on *fliZ*. (B) Reductions in pyroptosis and invasion in the Δ*metJ* mutant do not depend on *flhDC*. Because *flhDC* mutants are immotile, cells were infected and centrifuged at 500 × *g* for 10 min in order to promote host-bacterium interactions. For the assays whose results are presented in panels A and B, pyroptosis and invasion in 18592 LCLs were measured at 3 h postinfection using a modified gentamicin protection assay across at least three independent experiments. Percent cell death represents all 7-AAD^+^ cells under each infected condition, with the baseline uninfected cell death being subtracted. See the gating in Fig. 1A. (C) Reductions in motility in the Δ*metJ* mutant do not depend on *hilD*. Motility was measured after 6 h at 37°C on 0.3% LB agar. Data for panel C come from three biological replicates across two experiments. For panels A, B, and C, data were normalized to the global mean, and *P* values, indicated at the top, were calculated through a one-way analysis of variance with Sidak's multiple-comparison test.

If MTA acts through the flagellar regulon, we hypothesized that ablation of the flagellar regulon would prevent the further reduction of pyroptosis and invasion by MTA. However, deletion of *metJ* resulted in similar reductions in cell death and invasion regardless of the presence or absence of *fliZ* or *flhDC* (Fig. 6A and B). Therefore, the flagellar regulon is not necessary for the effects of MTA on pyroptosis and invasion.

If MTA acts through the SPI-1 pathway to suppress the flagellar regulon, we would expect *hilD* deletion to make the bacteria insensitive to the MTA-mediated suppression of motility. In contrast to this hypothesis, deleting *metJ* in wild-type and Δ*hilD* backgrounds resulted in the same decreases in motility (Fig. 6C). Therefore, MTA regulation of SPI-1 is not necessary for the effects of the metabolite on motility.

These findings are consistent with MTA independently regulating the SPI-1 pathway and motility regulon. This is in line with previous observations that while SPI-1 and flagellar gene expression are often correlated, few SPI-1 regulators control invasion by modulating the activity of the flagellar regulon and FliZ (46). We hypothesize that MTA suppresses SPI-1 and motility by interacting with a currently unknown factor upstream of both pathways. Importantly, if MTA interacts with a factor upstream of these virulence pathways, then other virulence pathways may also be suppressed by MTA.

### Disruption of bacterial methionine metabolism impairs virulence *in vivo*.

**FIG 7:**
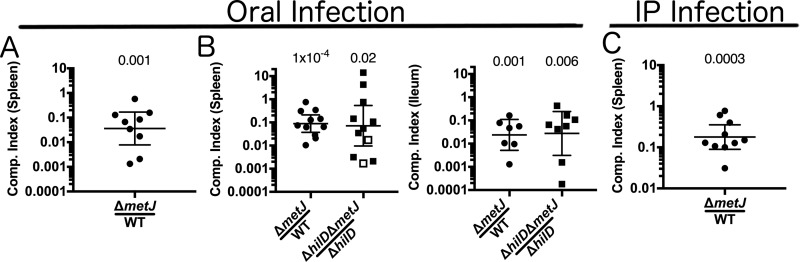
*metJ* deletion suppresses *S*. Typhimurium virulence *in vivo* independently of SPI-1. (A) *metJ* deletion reduced bacterial fitness in models of oral infection. C57BL/6J mice were infected with 10^6^ total *S*. Typhimurium bacteria from a 1:1 mixture of wild-type and Δ*metJ* bacteria by oral gavage. Spleens were harvested at 5 days postinfection, and bacteria were quantified to calculate the competitive (Comp.) index. (B) *metJ* deletion reduces bacterial fitness independently of SPI-1. C57BL/6J mice were infected with 10^9^ total *S*. Typhimurium bacteria from a 1:1 mixture of bacteria by oral gavage. Spleens and ileums were harvested at 3 days postinfection, and bacteria were quantified to calculate the competitive index. Open squares represent mice in which no Δ*hilD* Δ*metJ* bacteria were recovered. In these cases, the competitive index was set to be the maximum possible value by performing the calculation with one hypothetical Δ*hilD* Δ*metJ* colony. (C) *metJ* reduces bacterial fitness in intraperitoneal (i.p.) models of infection. C57BL/6J mice were infected with 10^3^ total *S*. Typhimurium bacteria from a 1:1 mixture of wild-type and Δ*metJ* bacteria by intraperitoneal injection. Spleens were harvested at 3 to 5 days postinfection, and bacteria were quantified to calculate the competitive index. The competitive index from each mouse is graphed as (number of Δ*metJ* mutant CFU/number of WT CFU)/(number of Δ*metJ* mutant CFU in the inoculum/number of WT CFU in the inoculum) or (number of Δ*hilD* Δ*metJ* mutant CFU/number of Δ*hilD* mutant CFU)/(number of Δ*hilD* Δ*metJ* mutant CFU in the inoculum/number of Δ*hilD* mutant CFU in the inoculum). *P* values, indicated at the top, were calculated by log transforming these ratios and comparing the value to an expected value of 0 using a one-sample *t* test. Data are from at least two independent experiments and are graphed using the geometric mean and 95% confidence interval.

Based on our findings that multiple virulence pathways are disrupted in the Δ*metJ* mutant, we hypothesized that the Δ*metJ* mutant would have impaired virulence *in vivo*. To test this, we orally coinfected C57BL/6J mice with wild-type and Δ*metJ* mutant *S*. Typhimurium and measured the bacteria's ability to infect and disseminate to the spleen. We found that the Δ*metJ* mutant had a 30-fold reduction in fitness compared to wild-type *S*. Typhimurium by 5 days postinfection (Fig. 7A).

As the Δ*metJ* mutant has reduced SPI-1 secretion, which is critical for Salmonella colonization and dissemination from the mouse gut (2, 8, 36, 49–51), we hypothesized that following oral inoculation, MTA-mediated suppression of SPI-1 was responsible for the reduced Δ*metJ* mutant fitness. To test this hypothesis, we knocked out the master SPI-1 regulator, *hilD*, in both the wild-type and Δ*metJ* mutant backgrounds to examine whether SPI-1 was necessary for the reduced Δ*metJ* mutant fitness *in vivo*. Unexpectedly, *S*. Typhimurium Δ*hilD* was still able to outcompete Δ*hilD* Δ*metJ* bacteria in the ileum and spleen at 3 days postinfection (Fig. 7B). There were no statistically significant differences between the competitive indexes calculated for the WT versus the Δ*metJ* mutant comparisons and the Δ*hilD* mutant versus the Δ*hilD* Δ*metJ* mutant comparisons in either tissue (*P* = 0.8 in the spleen and *P* = 0.9 in the ileum). This demonstrates that MTA suppresses *S*. Typhimurium virulence independently of its effects on SPI-1.

Our observation that MTA suppresses virulence in an oral model and in an SPI-1-independent manner prompted us to examine whether the Δ*metJ* mutant was attenuated through the intraperitoneal (i.p.) route as well. We observed a 5-fold reduction in the fitness of the Δ*metJ* mutant relative to that of the wild-type bacteria in the spleen (Fig. 7C). Importantly, the fitness cost was lower than that in spleens from mice orally infected with *S*. Typhimurium, which suggests that MTA suppresses fitness in an SPI-1-independent manner in the gut as well as by other mechanisms in systemic tissues. Together, these data demonstrate that MTA is able to regulate *S*. Typhimurium fitness at multiple stages of infection, highlighting the importance of the metabolite's regulation during infection.

### Disruption of methionine metabolism in *S*. Typhimurium reduces inflammatory cytokine production.

We previously reported that treatment of mice with MTA before infection with a lethal dose of *S*. Typhimurium resulted in reduced production of sepsis-related cytokines (interleukin-6 [IL-6] and tumor necrosis factor alpha [TNF-α]) and modestly prolonged survival (17). In light of our findings here, we hypothesized that the previously observed effects on inflammation may be due to MTA's impact on the microbe.

**FIG 8:**
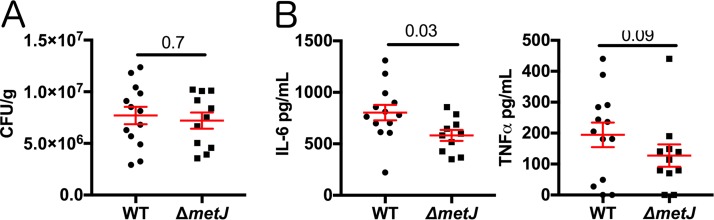
Bacterial methionine metabolism reduces host inflammation. (A) *metJ* deletion did not reduce bacterial fitness at 4 h after intraperitoneal infection. C57BL/6J mice were infected with 10^6^ wild-type or Δ*metJ* mutant *S*. Typhimurium bacteria. At 4 h postinfection, the spleens were harvested and the numbers of CFU were quantified. Data are graphed using the geometric mean and the 95% confidence interval. (B) *metJ* deletion reduced the host cytokine response to *S*. Typhimurium infection. Plasma harvested at 4 h postinfection showed reduced concentrations of the sepsis-associated cytokines IL-6 and TNF-α. Data were generated by ELISA. Error bars represent the standard error of the mean. All data represent those from three independent experiments normalized to the global mean, with each dot representing a biological replicate. *P* values, indicated at the top, for the number of CFU and IL-6 concentrations were calculated by an unpaired *t* test with Welch's correction. Because the TNF-α data are nonnormal, a Kolmogorov-Smirnov test was used to calculate the *P* value.

To test whether MTA reduced host inflammation by suppressing *S*. Typhimurium proinflammatory genes, we injected mice with a lethal dose (1 × 10^6^ CFU) of either wild-type or Δ*metJ* mutant *S*. Typhimurium by the intraperitoneal route and measured the numbers of CFU and cytokine levels at 4 h postinfection. Similar to what we observed with exogenous treatment of the mice with MTA (17), we did not see a difference in the numbers of CFU, demonstrating that both wild-type and Δ*metJ* mutant *S*. Typhimurium are equally capable of colonizing the spleen at this very early time point (Fig. 8A). In contrast, the IL-6 level was 28% lower (*P* = 0.03) in mice infected with the Δ*metJ* mutant (Fig. 8B). Further, TNF-α concentrations also showed a similar relative decrease (34%), though that result did not meet statistical significance (*P* = 0.09). Therefore, the Δ*metJ* mutant phenocopies the anti-inflammatory effects of treating mice with MTA before infection with *S*. Typhimurium. Therefore, the anti-inflammatory effects of MTA could be mediated not only through effects on the host but also through suppression of *S*. Typhimurium proinflammatory virulence factors.

## DISCUSSION

Here we report that exposing *S*. Typhimurium to exogenous MTA or increasing endogenous MTA production suppresses virulence *in vitro* and *in vivo*. This adds to a growing body of literature demonstrating that environmental factors can regulate critical Salmonella virulence factors (3–7, 52, 53). We found that MTA can suppress both SPI-1 and the flagellar regulon *in vitro*, as well as currently unidentified virulence factors *in vivo*. We hypothesize that this represents an example of host-pathogen cross talk, in which the host suppresses Salmonella virulence by increasing MTA concentrations. This would represent a novel antimicrobial mechanism and is supported by our findings that MTA plasma concentrations increase during infection (17). Future studies are needed to test how host modulation of MTA helps shape the outcome of infection at systemic sites as well as in the gut. Systemically, MTA decreases inflammatory cytokines, decreases the Salmonella burden, and, as we previously reported, modestly prolongs survival (17). In the gut, MTA could result in suppression of bacterial virulence or, alternatively, could help signal to the bacteria when and where it is appropriate to turn on the expression of key factors involved in virulence.

While no previous work examined the impact of *metJ* deletion on *S*. Typhimurium virulence, two papers reported the effects of Δ*metJ* deletion and virulence in other bacterial pathogens. Bogard et al. reported that Δ*metJ* deletion suppresses Vibrio cholerae virulence *in vivo* (54). Conversely, Cubitt et al. demonstrated that Δ*metJ* deletion increased the production of quorum sensing molecules and expression of virulence genes in the potato pathogen Pectobacterium atrosepticum (55). However, in both these cases, the metabolic changes responsible for these phenotypes are unknown. We hypothesize that our discovery of the role of *metJ* in regulating intracellular MTA concentrations could help explain these findings. If exogenous MTA can drive these phenotypes, similar to what we report here in *S*. Typhimurium, it would suggest that modulation of MTA concentrations represents a mechanism by which virulence can be manipulated across multiple bacterial species.

Our data support a model in which MTA serves as a regulatory signal that triggers the suppression of genes carried on SPI-1, the flagellar regulon, and other currently unknown virulence factors. Future genome-wide expression studies will help us determine if the effects of MTA on *in vivo* virulence are due to other known virulence determinants (such as SPI-2), genes involved in nutrient acquisition from the host, immune evasion, or other functions. One mechanism by which MTA may influence *S*. Typhimurium gene expression is by altering methylation. In prokaryotic and eukaryotic systems, SAM provides methyl for a variety of protein, DNA, and RNA methylation reactions (56–61). In eukaryotic systems, modulation of methionine metabolism resulting in changes to the cellular MTA and SAM pools can have important consequences on protein, DNA, and RNA methylation (62–65). Therefore, we hypothesize that increased MTA alters methylation to repress the expression or function of critical virulence factors.

Since exogenous and endogenous MTA affects Salmonella virulence, both bacterial and host methionine metabolism presents a therapeutic target. Previous studies tested MTA nucleosidase inhibitors against bacterial pathogens on the basis of the assumption that disrupting MTA nucleosidase would lead to MTA accumulation, resulting in an arrest of cellular growth and reduced bacterial viability (66–68). However, MTA nucleoside inhibitors showed, at most, modest bacteriostatic potential in these studies. In contrast, studies examining the effects of these compounds on quorum sensing also showed no changes in bacterial growth but did identify suppression of AI-2 synthesis (69, 70). This is in line with our observation that Salmonella growth is not impaired by increased concentrations of MTA in the Δ*metJ* mutant but that there are functional consequences on virulence. However, no study has examined the potential of these compounds to directly impact virulence independently of growth. Our data suggest that these compounds likely have antibacterial properties, because the disruption of methionine metabolism *in vivo* impairs virulence. Furthermore, other groups have developed *S*-methyl-5′-thioadenosine phosphorylase (MTAP) inhibitors, which block mammalian MTA catabolism (71–73), increasing MTA concentrations in tissues, plasma, and urine in murine models (74). Based on these results and our demonstration that high extracellular MTA concentrations suppress virulence, we hypothesize that MTAP inhibitors could be a host-directed therapy during Salmonella infection. Therefore, future studies will test whether MTA nucleosidase inhibitors and MTAP inhibitors could be harnessed to combat bacterial infections and improve clinical outcomes.

## MATERIALS AND METHODS

### Mammalian cells and bacterial strains.

HapMap LCLs were purchased from the Coriell Institute. LCLs and THP-1 monocytes were cultured at 37°C in 5% CO_2_ in RPMI 1650 medium (Invitrogen) supplemented with 10% fetal bovine serum (FBS), 2 μM glutamine, 100 U/ml penicillin G, and 100 mg/ml streptomycin. HeLa cells were grown in Dulbecco modified Eagle medium supplemented with 10% FBS, 1 mM glutamine, 100 U/ml penicillin G, and 100 mg/ml streptomycin. Cells used for Salmonella gentamicin protection assays were grown in antibiotic-free medium 1 h prior to infection.

All Salmonella strains are derived from the *S*. Typhimurium NCTC 12023 (ATCC 14028) strain and are listed in Table 1. All knockout strains were generated by bacteriophage lambda red recombination (75). Recombination events were verified by PCR, and the pCP20 plasmid was used to remove the antibiotic resistance cassette after recombination (76). Strains were cultured overnight in LB broth (Miller), subcultured 1:33, and grown for 2 h 40 min with shaking at 37°C before all experiments, unless otherwise noted. Strains containing the temperature-sensitive plasmid pKD46 or pCP20 were cultured at 30°C and removed at 42°C. Ampicillin was added to LB at 50 μg/ml, kanamycin at 20 μg/ml, and tetracycline at 12 μg/ml. Exogenous metabolites [5′-deoxy-5′-(methylthio)adenosine, l-phenylalanine, l-methionine, α-keto-γ-(methylthio)butyric acid sodium salt, adenosine, spermine, spermidine, and putrescine dihydrochloride (all from Sigma) and *S*-(5′-adenosyl)-l-methionine chloride (hydrochloride) (Cayman Chemicals)] were added to LB during the 2-h 40-min subculture step at a 300 μM final concentration unless otherwise noted. Infection of cells with metabolite-treated bacteria resulted in a greater than 1:50 dilution of the metabolite.

**TABLE 1:** Bacterial strains used in this study

Bacterial strain	Genotype	Plasmid	Resistance
DCK543	NCTC 12023 (ATCC 14028)		
DCK545	Δ*metJ*		
DCK546	NCTC 12023 (ATCC 14028)	pWSK29	Amp
DCK547	Δ*metJ*	pWSK29	Amp
DCK548	Δ*metJ*	pWSK29::*metJ*	Amp
DCK571	NCTC 12023 (ATCC 14028)	p67GFP	Amp
DCK573	Δ*metJ*	p67GFP	Amp
DCK574	NCTC 12023 (ATCC 14028)	pWSK129	Kan
DCK576	Δ*metJ*	pWSK129	Kan
DCK601	*fliZ*::Kan^r^		Kan
DCK604	Δ*metJ fliZ*::Kan^r^		Kan
DCK607	Δ*fliZ*		
DCK610	Δ*fliZ* Δ*metJ*		
DCK618	Δ*fliZ*	p67GFP	Amp
DCK620	Δ*fliZ* Δ*metJ*	p67GFP	Amp
DCK622	*flhDC*::Kan^r^		Kan
DCK623	Δ*metJ flhDC*::Kan^r^		Kan
DCK624	Δ*flhDC*		
DCK625	Δ*flhDC* Δ*metJ*		
DCK632	Δ*flhDC*	p67GFP	Amp
DCK633	Δ*flhDC* Δ*metJ*	p67GFP	Amp
DCK634	*hilD*::Kan^r^		Kan
DCK635	Δ*metJ hilD*::Kan^r^		Kan
DCK636	Δ*hilD*		
DCK637	Δ*hilD* Δ*metJ*		
DCK654	Δ*hilD*	pWSK29	Amp
DCK655	Δ*hilD* Δ*metJ*	pWSK129	Kan
DCK88	sspA::Tn*5* (*sipA* insertion)		Tet

### Gentamicin protection assay.

As previously described, inducible GFP plasmids were transformed into *S*. Typhimurium strains in order to assess both Salmonella-induced cell death and invasion by flow cytometry (24). Briefly, bacterial cultures were prepared as described above and used to infect LCLs and THP-1 monocytes (multiplicity of infection [MOI], 10 or 30), as well as HeLa cells (MOI, 5). For experiments with nonmotile Δ*flhDC* mutants, cells were centrifuged at 500 × *g* for 10 min to enable infection. At 1 h postinfection, cells were treated with gentamicin (50 μg/ml), and IPTG (isopropyl-β-d-thiogalactopyranoside) was added at 2 h postinfection to induce GFP expression. At 3.5 h postinfection, cells were assessed for cell death using 7-aminoactinomycin D (7-AAD; Biomol), with death being read by a Guava EasyCyte Plus flow cytometer (Millipore). Percent invasion was determined by quantifying the number of GFP^+^ 7-AAD^−^ cells at 3.5 h postinfection using the Guava EasyCyte Plus flow cytometer. Gates were set by using uninfected cells to gate out GFP-negative (GFP^−^) cells and by using the natural break in 7-AAD^−^ and 7-AAD-positive (7-AAD^+^) cells (Fig. 1A).

### Metabolomics.

Bacteria were grown overnight as described above, subcultured 1:33 in 10 ml LB, and grown for 2 h 40 min. After thorough washing in phosphate-buffered saline (PBS), samples were flash frozen and thawed, and 0.5 ml PBS was added directly onto the pellets. Samples were then transferred to 2 ml CK01 bacterial lysis tubes (Bertin). These were then taken through 3 cycles of 20-s bursts at 7,500 rpm with 30-s pauses in between bursts using a Bertin Precellys homogenizer (the protocol recommended by Bertin). Samples were spun at 5,000 × *g* for 5 min, and a Bradford assay was performed on each lysate to gather protein concentration values. One hundred microliters from each homogenate was pipetted directly into a 2-ml 96-well plate (Nunco).

The internal standard methanol solution was made by pipetting 166.7 μl of NSK-A standard (Cambridge Isotope) at 500 μM, 62.5 μl of 500 nM d3-MTA, and 49.771 ml of methanol (MeOH). Nine hundred microliters of this internal standard solution in MeOH was pipetted into all of the standard and sample wells. The plate was then capped and mixed at 700 rpm at 25°C for 30 min. The plate was then centrifuged at 3,000 rpm for 10 min. Using an Integra Viaflo96 pipette, 600 μl of extract was pipetted out and transferred to a new 96-well plate. The extracts were allowed to dry under a gentle stream of nitrogen until completely dry. Thirty-two microliters of 49:50:1 water-acetonitrile-trifluoroacetic acid was added to each well and mixed at 650 rpm for 10 min at room temperature. Then, 128 μl of 1% trifluoroacetic acid was added to each well, the contents were mixed briefly, and the plate was centrifuged down to give a total of 160 μl of sample.

The samples were analyzed using ultraperformance liquid chromatography-electrospray ionization-tandem mass spectrometry (UPLC-ESI-MS/MS) using a customized method allowing chromatographic resolution of all analytes in the panel. Flow from the liquid chromatography separation was introduced via positive-mode electrospray ionization (ESI^+^) into a Xevo TQ-S mass spectrometer (Waters) operating in multiple-reaction-monitoring (MRM) mode. MRM transitions (compound-specific precursor-to-product ion transitions) for each analyte and internal standard were collected over the appropriate retention time. The data were imported into Skyline software (https://skyline.gs.washington.edu/) for peak integration and exported into Excel software for further calculations.

### Bacterial RNA isolation and qPCR.

Bacteria were grown as described above, and RNA was isolated from 5 × 10^8^ bacteria using the Qiagen RNAprotect Bacteria reagent and an RNeasy minikit (Qiagen) according to the manufacturer's instructions. RNA was treated with DNase I (NEB), and 500 ng was reverse transcribed using an iScript cDNA synthesis kit (Bio-Rad Laboratories). qPCR was performed using the iTaq Universal SYBR green Supermix (Bio-Rad Laboratories). Ten-microliter reaction mixtures contained 5 μl of the supermix, a final concentration of 500 nM each primer, and 2 μl of cDNA. Reactions were run on a StepOnePlus real-time PCR system (Applied Biosystems). The cycling conditions were as follows: 95°C for 30 s, 40 cycles of 95°C for 15 s and 60°C for 60 s, and 60°C for 60 s. A melt curve was performed in order to verify single PCR products. The comparative threshold cycle (*C_T_*) method was used to quantify transcripts, with the ribosomal *rrs* gene serving as the endogenous control. Δ*C_T_* values were calculated by subtracting the *C_T_* value of the control gene from the *C_T_* value of the target gene, and the ΔΔ*C_T_* value was calculated by subtracting the wild-type Δ*C_T_* value from the mutant Δ*C_T_* value. Fold change represents 2^−ΔΔ*CT*^. Experiments included three technical replicates, and the data represent the qPCR results from five separate RNA isolation experiments. The oligonucleotides used are listed in Table 2.

**TABLE 2:** Oligonucleotides used in this study

Primer purpose and gene or plasmid	Direction and use^*a*^	Sequence	Reference
qPCR			
*hilA*	Fwd	ATAGCAAACTCCCGACGATG	79
	Rev	ATTAAGGCGACAGAGCTGG	
*hilD*	Fwd	GGTAGTTAACGTGACGCTTG	79
	Rev	GATCTTCTGCGCTTTCTCTG	
*rtsA*	Fwd	ACCCGTGGTGAGCTTGATGAGT	79
	Rev	CCTGTCCAGGTGGGGAGCAT	
*sicP*	Fwd	AGATGATATCTGGTTATTGAACGGTATG	79
	Rev	CTGCCGCCAGATAGAATCG	
*hilC*	Fwd	CTCACCTCTTCAGCGGCCAGT	79
	Rev	CACCCGCAAATGGTCACAGGCT	
*prgH*	Fwd	TGAACGGCTGTGAGTTTCCA	79
	Rev	GCGCATCACTCTGACCTACCA	
*sipB*	Fwd	GGCGCGCTGCTAACCAT	79
	Rev	TCGCCCCACCGGTAAAA	
*invF*	Fwd	TGTCGCACCAGTATCAGGAG	80
	Rev	AAATAGCGCGAAACTCAGGA	
*hilE*	Fwd	AAAGCCGGATCAAAGGTTTT	80
	Rev	CTTTCACCGTTTTCCCGTTA	
*flhD*	Fwd	TGTTCCGCCTCGGTATCAAC	81
	Rev	CGCGAATCCTGAGTCAAACG	
*fliA*	Fwd	GATTGAATCGCTGCCGGAAC	81
	Rev	ACTATGCAACTGGCTGACCC	
*fliZ*	Fwd	AAACATTTCCCACGATCTGC	81
	Rev	CGGTAAAGGGGGATTTCTG	
*rrs*	Fwd	CGGGGAGGAAGGTGTTGTG	80
	Rev	CAGCCCGGGGATTTCACATC	
Bacteriophage lambda Red deletion			
*metJ*	Fwd cassette generation	GGGCTCAGGTTCAGACCTCAATATTAATGACGAAGAGGATTAAGTATCTCGTGTAGGCTGGAGCTGCTTC	
	Rev cassette generation	GAATCGTTAAAAAAGCGCGGCCAGAGGCGTTCTGACCGCATGCTTTGCTACATATGAATATCCTCCTTAG	
	Upstream	CATCTGCGACCGCTAACTT	
	Downstream	TTTATCCACCGAGGGTTATTCG	
*fliZ*	Fwd cassette generation	CGAAAAGTGCCGCACAACGTATAGACTACCAGGAGTTCTCGTGTAGGCTGGAGCTGCTTC	
	Rev cassette generation	CACGTTTCACCAACACGACTCTGCTACATCTTATGCTTTTCATATGAATATCCTCCTTAG	
	Upstream	CATCGAACTGGTGACTGAAGA	
	Downstream	CTACAGCCATTACTCCCATCAG	
*flhDC*	Fwd cassette generation	GTGCGGCTACGTCGCACAAAAATAAAGTTGGTTATTCTGGGTGTAGGCTGGAGCTGCTTC	
	Rev cassette generation	ATGACTTACCGCTGCTGGAGTGTTTGTCCACACCGTTTCGCATATGAATATCCTCCTTAG	
	Upstream	CGAGTAGAGTTGCGTCGAATTA	
	Downstream	ATCCTTCCGCTGTTGACTATG	
*hilD*	Fwd cassette generation	CCAGTAAGGAACATTAAAATAACATCAACAAAGGGATAATGTGTAGGCTGGAGCTGCTTC	
	Rev cassette generation	TTAATAAAAATCTTTACTTAAGTGACAGATACAAAAAATGCATATGAATATCCTCCTTAG	
	Upstream	CTGGGCTTGTTATCGTCTTCT	
	Downstream	CAGGAGGGTTATGAGCAGTATC	
pKD46	Fwd	CAGTCATAGCCGAATAGCCT	75
pKD46	Rev	CGGCCACAGTCGATGAATCC	75

a Fwd, forward; Rev, reverse.

### Analysis of bacterial protein expression.

Bacteria were grown as described above. For analysis of cell lysates, bacterial cultures were centrifuged at 10,000 × *g* for 5 min. The supernatant was discarded, and the pellets were lysed in 2× Laemmli buffer (Bio-Rad) with 5% 2-mercaptoethanol. Samples were boiled for 10 min and analyzed on Mini-Protean TGX stain-free gels (Bio-Rad). Bands were stained with a rabbit anti-SipA antibody overnight at 4°C. Antibodies were then detected by staining with the Li-Cor IRDye 800CW donkey anti-rabbit immunoglobulin antibody. SipA was quantified using a Li-Cor Odyssey Fc imaging system paired with Image Studio software. Bands were normalized to total protein using a TGX stain-free system. Total protein was quantified with Fiji software (77).

For secreted protein analysis, cultures were centrifuged at 10,000 × *g* for 5 min, and supernatants were passed through a 0.2-μm-pore-size syringe filter. At this point, 6 μl of 100-ng/μl bovine serum albumin (BSA) was added to 600 μl of supernatant as a loading control. Chilled 100% trichloroacetic acid (TCA) was added to a final concentration of 10%, and the mixture was incubated on ice for 10 min. Six hundred microliters of chilled 10% trichloroacetic acid was added, and the solution was incubated on ice for another 20 min before being centrifuged at 20,000 × *g* for 30 min. Pellets were washed twice with acetone and resuspended in 2× Laemmli buffer (Bio-Rad) with 5% 2-mercaptoethanol before boiling for 10 min. Proteins were then analyzed as described above.

### Motility assay.

The motility assay was performed as previously described (35). Briefly, strains were cultured overnight in LB broth (Miller), subcultured 1:33, and grown for 2 h 40 min with shaking at 37°C. Two microliters of the subcultured solution was plated in the center of a 0.3% agar LB plate supplemented with 50 μg/ml ampicillin. Metabolites or DMSO was added to the solution prior to the agar solidifying in order to allow exposure of the bacteria to the metabolite for the entirety of the assay. The plates were incubated at 37°C for 6 h before the halo diameter was quantified.

### Mouse infection studies.

Mouse studies were approved by the Duke Institutional Animal Care and Use Committee and adhere to the guidelines in the *Guide for the Care and Use of Laboratory Animals* of the National Research Council (78). Bacteria were grown as described above, washed, and resuspended in PBS. Inocula were confirmed by plating for determination of the number of CFU. For oral infections, age- and sex-matched 7- to 16-week-old C57BL/6J mice were fasted for 12 h before infection. At 30 min prior to infection, mice received 100 μl of a 10% sodium bicarbonate solution by oral gavage. Age- and sex-matched mice were then infected with 10^6^ bacteria in 100 μl PBS by oral gavage. At 5 days postinfection, mice were euthanized by CO_2_ asphyxiation, and spleens and ileums were harvested, homogenized, weighed, and plated on LB agar containing either ampicillin or kanamycin. For Δ*hilD* mutant experiments, mice were infected with 10^9^ bacteria as described above and harvested 3 days postinfection.

For intraperitoneal (i.p.) competitive index experiments, age- and sex-matched 6- to 20-week-old C57BL/6J mice received 10^3^ bacteria in 100 μl of PBS by i.p. injection. At 3 to 5 days postinfection, mice were euthanized by CO_2_ asphyxiation, blood was drawn by cardiac puncture, and spleens were harvested, homogenized, weighed, and plated on LB agar containing ampicillin or kanamycin.

The competitive index was calculated as (number of Δ*metJ* mutant CFU/number of WT CFU)/(number of Δ*metJ* mutant CFU in the inoculum/number of WT CFU in the inoculum). Statistics were calculated by log transforming this ratio from each mouse and comparing the value to an expected value of 0 using a one-sample *t* test. Differences between competitive indexes were calculated by performing a Student's *t* test comparing the log-transformed competitive indexes.

For high-dose i.p. injection, age- and sex-matched 6- to 8-week-old C57BL/6J mice received 10^6^ bacteria in 100 μl of PBS by i.p. injection. At 4 h postinfection, mice were euthanized by CO_2_ asphyxiation, blood was drawn by cardiac puncture, and spleens were harvested, homogenized, weighed, and plated on LB agar containing ampicillin. Plasma was isolated using plasma separation tubes with lithium heparin (BD). IL-6 and TNF-α were then quantified from plasma and spleen extracts using DuoSet enzyme-linked immunosorbent assay (ELISA) kits (R&D Systems).
